# Social and Activity Participation and Subjective Well-Being Among Older Adults in Japan: A Comparative Analysis of Healthy and Care-Needing Groups

**DOI:** 10.1155/jare/5990506

**Published:** 2025-07-28

**Authors:** Jianyu Huang, Ziyan Wang, Richard Ssempala

**Affiliations:** ^1^Graduate School of Contemporary System Sciences, Osaka Metropolitan University, Osaka, Japan; ^2^Graduate School of Economics, Osaka Metropolitan University, Osaka, Japan; ^3^Department of Economic Theory and Analysis, School of Economics, Makerere University, Kampala, Uganda

**Keywords:** care-needing, Japan, older adults, social and activity participation, subjective well-being

## Abstract

**Objectives:** This study explores the relationship between social and activity participation (SAP) and subjective well-being (SWB) among older adults in Japan, with a particular focus on differences by care-needing status.

**Methods:** Data were drawn from the 2019 Sakai City Older Adults Survey, including 5469 healthy and 406 care-needing individuals aged 65 and over (65.59% female). An ordered probit model was used to estimate the association between SAP and SWB, which was measured on an 11-point scale and categorized into three levels. To address potential endogeneity, instrumental variable (IV) analysis was conducted using information source diversity and health consciousness as instruments.

**Results:** Higher frequency of SAP was positively associated with SWB among both healthy and care-needing older adults. While the association was stronger and more consistent among the healthy group, IV analysis revealed a statistically significant and robust relationship among care-needing individuals as well. The findings suggest that even older adults facing physical or cognitive limitations may experience psychological benefits from SAP.

**Conclusions:** SAP is associated with better SWB among older adults, including those with care-needing status. These results support the promotion of inclusive community-based programs to enhance psychosocial health in aging populations.

## 1. Introduction

Japan's population is aging rapidly. Currently, one in every three people is over 65 years old [1–3], and the proportion of older adults is projected to reach nearly 40% by 2040, according to national demographic forecasts [4].

Moreover, with the declining birthrate and aging population, older adult care and welfare have become a major issue in the context of dramatic changes in the country's social functions and family structure. Japan' s 2000 Long-Term Care Insurance (LTCI) system aimed to rationalize aging-related care by shifting it away from medical institutions [5].

However, the government's concern about the soaring cost of long-term care has been raised as it reevaluates LTCI sustainability [5]. In 2006, the government implemented measures aimed at identifying care-needing or prefrail older adults and providing early preventive care programs to reduce functional decline and delay dependence on long-term care. However, low participation persists due to accessibility and awareness issues, prompting policy shifts toward promoting social and activity participation (SAP) to reduce isolation and prevent disability [6, 7]. With extensive efforts for disability prevention after the implementation of the LTCI system, public long-term disability prevention plans now focus on promoting SAP and preventing the isolation of older people, as isolation is a well-documented risk factor for both disability and mortality [7–9].

Accordingly, the present study aims to examine how participation in SAP influences the subjective well-being (SWB) of older adults. SWB, as a key indicator of psychological resilience, plays a protective role in mental and physical health. Previous research has shown that lower levels of SWB increase the risk of depression and accelerate functional decline among older adults [10–12]. Conversely, enhancing SWB may contribute to healthier aging trajectories and delay the need for long-term care. Therefore, identifying effective forms of SAP that promote SWB is essential for the development of preventive care strategies within Japan's comprehensive community care system [3, 13, 14]. Building on this policy relevance, the present study focuses on the role of social and SAP in shaping the SWB of older adults. By analyzing whether and how engagement in SAP contributes to psychological well-being, the study aims to provide empirical evidence that can inform community-based preventive care strategies. The analysis draws on a unique individual-level dataset from Japan, encompassing residents aged 65 and over. Particular attention is given to two distinct groups: those not requiring any regular nursing care (*n* = 5469), and those currently in need of care (*n* = 406), reflecting both healthy and care-needing groups segments of the older population [15].

### 1.1. SAP and SWB

SAP has long been associated with improved well-being among older adults [16, 17]. Two commonly used terms in the literature are “SAP” and “social participation.” The latter generally refers to interpersonal or community-based activities such as volunteering or neighborhood involvement. While definitions vary [18], this study adopts the term SAP to encompass not only conventional forms, such as volunteering and community involvement, but also broader dimensions, including cultural, preventive, and productive activities, such as gainful employment. This expanded definition reflects the evolving understanding of civic engagement among older adults and corresponds to the eight activity categories identified (see Appendix A) in the Sakai City Older Adults Survey.

A growing number of studies have shown that SAP is positively associated with SWB, positively with life satisfaction and positive affect, and negatively associated with negative affect [19–21]. Despite growing evidence of SAP's positive effects on health and SWB, several questions remain concerning its impact on older adults. First, scholars are currently seeking to elucidate whether and how participation in different types of activities is associated with positive outcomes in later life [22–26]. Although research on various activity types and later-life health is expanding, few studies examine these activities concurrently [27, 28], a major gap in the literature [21, 25, 29, 30]. This hinders the understanding of which activities are more or less associated with health and well-being outcomes for older adults, and whether the type of activity and frequency of participation have the same effect on the SWB of different older adult groups.

Whether care-needing individuals can similarly benefit from participation remains an unresolved question in the literature. Addressing this knowledge gap is essential for designing inclusive interventions in rapidly aging societies, where functional limitations and social exclusion are becoming increasingly common [12, 31].

## 2. Methods

### 2.1. Data and Sample

This study utilizes the “Sakai City Older Adults Survey” (December 2019), which provides individual data on older adults' living conditions, health, and welfare (Health and Welfare Bureau of Sakai, 2019). A total of 9400 residents aged 65 and over were randomly selected, yielding 6181 valid responses. After removing incomplete data, 5986 samples remained, including 5469 healthy older adults and 406 requiring care. Participants were categorized into two groups based on their responses to the questionnaire item: “Do you require any assistance or care in daily life?” Individuals who selected “I do not require any assistance or care” were classified as healthy, while those who responded “I currently receive some form of care” (including informal care without official certification) were classified as the care-needing group. This classification aligns with the Japanese long-term care context, where the need for care, regardless of formal LTCI status, indicates potential physical or cognitive limitations. The distinction between these groups is crucial, as care-needing individuals often face mobility limitations that restrict their participation in social activities, unlike their healthier counterparts who actively engage in community life [32].

The survey included 12 key demographic and socioeconomic variables, such as age, gender, marital status, housing, financial status, family structure, and nursing care status. SAP was classified into eight types: volunteer groups, sports clubs, interest-based groups, cultural circles, disability prevention programs, senior citizens' clubs, local councils, and gainful employment. The dataset used in this study is not publicly available, but detailed information and the official overview of the Sakai City Older Adults Survey (2019) can be found on the Sakai City website (in Japanese): https://www.city.sakai.lg.jp/shisei/gyosei/shishin/fukushi/kourei-kaigo_keikaku/75402820211117160542577.html Researchers may request access by submitting a formal application to the Sakai City Longevity Support Division.

### 2.2. Variable and Model Selection

The main dependent variable, SWB, was assessed using an 11-point self-reported scale (0 = “very unhappy” to 10 = “very happy”), based on Item 8 (3) of the Sakai City Older Adults Survey. This aligns with the Organization for Economic Co-operation and Development (OECD) [33] and World Happiness Report standards. In a Eurostat expert report using European Union Statistics on Income and Living Conditions data, SWB (life satisfaction) was categorized using the 0–5/6–8/9–10 scheme as low/medium/high satisfaction, specifically for comparability reasons between countries. This supports the use of identical thresholds in our study [34].

**Figure 1:**
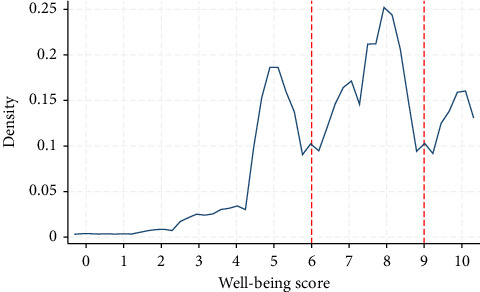
Kernel density estimate of well-being score.

To visually examine the empirical distribution of SWB scores and justify the choice of cutoff points, we plotted a kernel density estimate of the SWB variable (Figure 1). As shown in the figure, the distribution is bimodal, with two clear local peaks and troughs around scores of 6 and 9. These troughs serve as natural breakpoints for categorizing SWB into low (0–5), medium (6–8), and high (9–10), in alignment with the Eurostat practice [34].

This study first used the ordered probit model to estimate the latent variable underlying SWB, which is a subjective and abstract conceptual indicator that is often difficult for individuals to accurately judge; therefore, this latent variable was accurately stratified on a scale of 0–10. Due to the possible reliability bias of individual heterogeneity [35], we further stratified the SWB variable, following Eurostat's established approach [35].

(1) $$\begin{array}{c} s^{*}=\left\{\begin{array}{c} 1\;y_{i}^{*}<5,\\ 2\;5\leq y_{i}^{*}\leq 8,\\ 3\;y_{i}^{*}>8. \end{array}\right. \end{array}$$

Suppose *F*(·) is a standard that is normally distributed, then *α*_1_ and *α*_2_ are new threshold values. The independent variable vector *x* includes SAP, with *β* being the corresponding estimated coefficient. Treating *s*^∗^ as the dependent variable, the log-likelihood function of the ordered probit model is formulated as follows:

(2) $$\begin{array}{c} \ln\left(\beta ,\alpha_{1}{,\alpha }_{2},\alpha_{3}\right)=\overset{n}{\underset{i=1}{\sum }}\overset{3}{\underset{j=1}{\sum }}1\left\{s^{*}=j\right\}\ln\left[F\left(\alpha_{2}-x_{i}^{\prime }\beta \right)-F\left(\alpha_{1}-x_{i}^{\prime }\beta \right)\right], \end{array}$$

where 1{*·*} is the demonstrative function that takes the value of 1 when the condition in parentheses holds, and 0 otherwise. By maximizing this log-likelihood function, the coefficient *β* and parameters *α*_1_ and *α*_2_ in the ordered response model can be estimated.

The frequency of activity participation was defined by six levels: no participation, several times a year, 1–3 times a month, once a week, 2–3 times a week, and 4 or more times a week. The dummy variables for SAP frequency were set as 1 for those who participated in any of the 8 types of activities 1–3 times a month or more, and 0 otherwise, as shown in Table 1. The details of the eight types of activities, their frequency, and the variable definitions are provided in Appendix A.

Other demographic variables were also assessed, including the number of activities participated in, financial status, age-specific variables, level of health awareness, marital status, family structure, gender, property ownership, number of channels of information access, instrumental activities of daily living (IADL), body mass index (BMI), and neighborhood strength (Table 1).

Due to physical limitations, care-needing older adults may face constraints in participating in social activities; therefore, this study also takes into account the potential endogeneity of SAP within the care-needing older adult sample. To address potential endogeneity of SAP, two instrumental variables (IVs) were introduced in the questionnaire: (a) the number of information sources, derived from Question 5 (9), “Where do you get information about your daily life?” (a multiple-choice item), and (b) health consciousness, based on the Question 8 (2), “Are you conscious about maintaining and improving your health?.” Although it is plausible that general awareness of health and access to diverse information may reflect individual characteristics, there is no theoretical or empirical basis to suggest that these factors directly influence SWB, except through their effects on SAP. Thus, the exclusion restriction is reasonably maintained. Prior studies also support that health consciousness and information access are positively associated with participation in social and community activities [18, 20], providing additional justification for the use of these variables as instruments.

## 3. Results and Analysis

### 3.1. Basic Regression Results

This study uses the ordered probit model to examine the effect of SAP on enhancing older adults' SWB, using it as the baseline regression model. SWB is also treated as a continuous variable and analyzed using the ordinary least squares (OLS) regression method. As mentioned before, the results from the tests of these two models show a high degree of consistency in the sign and significance of the coefficients, except for the difference in the estimated magnitudes of the estimated coefficients.

The empirical results of models (1) and (2) in Table 2 show that SAP significantly improves the SWB of healthy older adults who do not require care. This effect is significantly stronger, when the effect of the number of types of activities participated in is excluded from the results of models (3) and (5). However, the current results show that SAP is not significantly associated with higher SWB among care-needing older adults who require care. A possible explanation is that due to limited physical mobility, their participation in social activities may be significantly constrained. Therefore, the key IVs in the model may suffer from endogeneity, which warrants further examination within this study.

Economic status, being married, female, good neighborly relations, and housing ownership are consistently positive predictors of SWB in the healthy sample. Only gender and advanced age (over 75) were significant among care-needing individuals. These findings suggest that the benefits of SAP for SWB are more prominent among healthier older adults, while care-needing status may limit both the extent and psychological returns of such engagement.

The estimated coefficients of the ordered probit model did not reveal comprehensive information; only the significance and sign of the regression coefficients revealed whether SAP affects the SWB of older adults and the direction of the effect. Therefore, the marginal utility of SAP was further analyzed. The marginal effects shown in Table 3 indicate that, among healthy older adults who do not require care, those who participate in SAP are 7.5% times more likely than others to report a higher level of well-being. Those involved in more than two types of SAP were 1.9% times less likely than others to report lower levels of SWB. The marginal effect of SAP increased, when the number of types of SAP was excluded, as shown in Table 3.

Table 4 presents the marginal effects of each activity type on SWB among all older adults in the sample. Participation in sports groups or clubs, groups with related interests, and gainful employment significantly increased the probability of reporting higher levels of SWB while reducing the probability of reporting lower levels. For example, participation in groups with related interests increased the probability of reporting high SWB by 7.3% times and decreased the likelihood of reporting low SWB by 2.6% times. In contrast, other activity types such as participation in cultural circles, senior citizen clubs, or town councils did not exhibit statistically significant effects. These results indicate that not all types of activity participation contribute equally to well-being, and some may be more psychologically rewarding than others.

**Figure 2:**
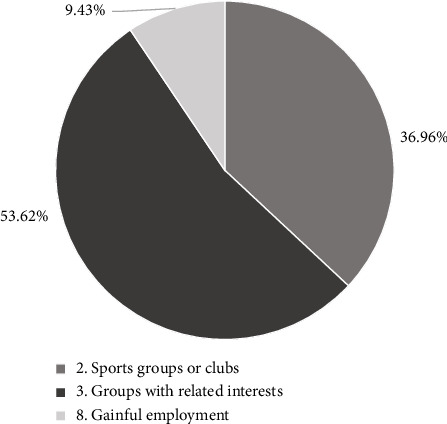
Contribution of activity types to SWB (Shapley decomposition) (note: Shapley value decomposition shows the relative contribution of three statistically significant activity types to SWB variance in an OLS model. Percentages represent the share of total *R*^2^ = 0.027).

To further validate the marginal effects discussed above, a Shapley value decomposition based on the OLS model was applied to estimate the relative contribution of each activity type with statistically significant effects (Figure 2). Among the three significant activities, participation in groups with related interests accounted for the largest share of explained variance in SWB (53.62%), followed by participation in sports groups or clubs (36.96%), and gainful employment (9.43%). This result underscores the particularly strong role of interest-based social engagement in promoting well-being in later life.

### 3.2. Endogeneity Treatment

As noted above, it is necessary to consider the potential endogeneity of SAP among care-needing older adults. For instance, some individuals may be willing to invest time in SAP for higher-level psychological fulfillment, yet their physical limitations restrict their ability to participate. If such a situation exists, it would introduce endogeneity and undermine the robustness of the results. For this reason, the issue of endogeneity needs to be further discussed and analyzed. In order to identify and eliminate endogeneity in the possible reverse causality between SAP and SWB in older adults, appropriate IVs need to be recruited for SAP, which should be further tested using two-stage least squares (2SLS) regression.

To verify the validity of the IVs, this study conducted a first-stage regression based on models (7-1) and (8-1) in Table 5. The F-statistic exceeds the conventional threshold of 10, indicating that the instruments are sufficiently strong to avoid weak instrumental bias. When the (a) number of information sources and (b) health consciousness were introduced, the estimated coefficients of the two variables were significantly associated with SAP. Based on this, this study considered the number of information sources and health consciousness to be two valid IVs.

A 2SLS regression was conducted on older adults' SWB using these two IVs: (a) number of information sources, (b) health consciousness, whose results are presented in Table 5. The regression results in Table 5 show that the SWB of the group of older adults needing care is significantly affected by SAP, and the estimated coefficient increases substantially. However, the possibility of monotonicity violations should be acknowledged, that is, the IVs may influence participation heterogeneously across subpopulations, which could inflate the local average treatment effect (LATE). This limitation suggests that the causal interpretation should be restricted to compliers, meaning individuals whose SAP is influenced by the IVs.

A comparison between the estimation results of model (7) and model (1) yields a Hausman test *p* value of 0, indicating a systematic difference between the two regression results. Similarly, the Hausman test comparing model (10) and model (2) for both healthy and care-needing older adults also yields *p* values of 0, suggesting the presence of endogeneity in both models. This study conducted the Hansen J overidentification test to assess the validity of the instruments. For both subsamples, the test fails to reject the null hypothesis that the instruments are uncorrelated with the error term (*p* > 0.1), suggesting that the IVs are exogenous and the overidentifying restrictions are valid.

While the Hansen test supports instrument validity, potential violations of exclusion restrictions warrant cautious interpretation. This study adopts the approach proposed by Conley et al. [36], which allows for plausible deviations from the strict exogeneity assumption, to examine how the estimated coefficients change when the instruments are not fully exogenous.

Specifically, we allowed the number of information sources and health consciousness to have potential direct effects on SWB up to 0.03 and 0.05, respectively. Among care-needing older adults (i.e., those in need of care), the 95% union confidence interval for the coefficient of SAP ranges from 0.024 to 1.624, which lies entirely above zero. This suggests that even under modest deviations from the exclusion restriction, the positive association between participation and SWB remains statistically significant and robust. Among healthy older adults (i.e., those not in need of care), the UCI interval is even tighter, ranging from 0.825 to 2.087, further supporting the credibility of the observed relationship.

## 4. Discussion and Future Directions

This study contributes to the growing body of literature on aging by not only examining the association between SAP and SWB but also by addressing potential endogeneity concerns through the use of IV techniques. The findings indicate that while SAP is positively associated with SWB among healthy older adults, the strength and robustness of this association are even more pronounced among care-needing older adults when potential endogeneity is statistically accounted for. This challenges conventional assumptions that care-needing older adults are passive participants with limited capacity to benefit from SAP [10, 27].

Both the ordered probit and OLS results consistently show that the frequency and diversity of SAP are positively associated with higher levels of SWB among healthy older adults. In contrast, the associations observed among care-needing individuals were initially weak and statistically insignificant, likely due to selection bias and lower participation rates. However, after applying 2SLS estimation with two theoretically motivated instruments, namely, the number of information sources and health consciousness, the strength of the association between SAP and SWB among care-needing older adults became statistically significant and substantively meaningful. These results suggest that prior research may have underestimated the potential benefits of SAP for SWB in physically vulnerable older populations [23, 28].

Marginal effects from the ordered probit models further support this conclusion. Among healthy older adults, frequent participation in SAP is associated with a 7.5% times increase in the likelihood of reporting high SWB. Even among care-needing individuals, engaging in multiple types of SAP substantially reduces the likelihood of low SWB. These findings reinforce existing evidence that active engagement can enhance the quality of life and delay physical or cognitive decline [24, 25, 37]. While the overall frequency and diversity of participation are positively associated with SWB, the marginal effect analysis further reveals that not all types of activity participation are equally effective. Among the eight activity types examined, participation in sports groups, interest-based groups, and gainful employment showed statistically significant effects in increasing the probability of reporting high SWB. To further examine their relative importance, a Shapley value decomposition was conducted. The results indicate that interest-based groups account for over half of the explained variance among the three, suggesting that psychologically meaningful and self-selected social engagement may have the greatest impact on well-being. This finding aligns with theories of autonomy and intrinsic motivation in late-life engagement and emphasizes the need to consider both quantity and quality of social participation opportunities when designing interventions for older adults.

The demographic analysis underscores several key predictors of SWB: being female, married, financially secure, and having strong neighborhood ties, which are all positively associated with higher well-being. These results are consistent with previous findings on the role of economic and social capital in promoting life satisfaction in later life [7, 26]. Interestingly, individuals aged 75 and above (the “super-aged”) also report relatively higher SWB, which may reflect psychological resilience or successful adaptation to aging [16].

Nonetheless, several limitations of this study should be acknowledged. First, the cross-sectional nature of the data limits our ability to infer long-term effects or dynamic changes in SAP and SWB over time. While IV estimation can address endogeneity to some extent, it is not a complete substitute for longitudinal data. Second, the generalizability of the results may be constrained by the focus on a single Sakai city, Japan, despite its demographic similarity to the national average. Regional variations in community infrastructure or cultural attitudes toward aging may lead to different outcomes elsewhere [6]. Third, while our instruments passed validity and strength tests, the possibility of monotonicity violations or LATE bias remains, especially given the heterogeneity in participation behavior. Finally, the current study focuses on traditional forms of in-person participation, without addressing newer forms of digital or virtual engagement that may become increasingly relevant, particularly in postpandemic contexts [12].

Prior research has indicated that the use of information and communication technology (ICT) can play a positive role in reducing social isolation among older adults and may act as a substitute for certain forms of in-person SAP [38]. As part of the integrated care agenda, it is beneficial to consider alternative governance models, including government, local authorities, and private-sector approaches. It is also necessary to discuss the enabling role of emerging technologies in care projects, such as social–ecological survival probability [39], artificial intelligence and internet of things–based healthcare programs for older adults [40], immersive virtual reality environments [41], and other digital innovations. Future research should aim to incorporate longitudinal data, explore the role of digital SAP, and examine how regional policy differences influence the effectiveness of community-based interventions. Such extensions would offer a more comprehensive understanding of how to support active aging in diverse and evolving social environments.

## 5. Conclusions and Future Directions

This is one of the few studies to examine the positive outcomes of care-needing older adults who need care and to identify the eight types of SAP that significantly enhance SWB in Sakai City, Japan. From the results, it appears that the role of SAP may have been underestimated [27, 28]. The findings indicate that active participation in SAP promotes health and well-being across different types and frequencies of activity, suggesting that the health benefits lie more in the engagement itself than in specific social contexts [18, 28, 29]. The study also identified effective facilitators of SAP for care-needing older adults.

Japan's community-based integrated care system aims to address the challenges of population aging by promoting preventive strategies such as SAP [1, 5, 14]. However, disparities in access persist, particularly for care-needing older adults in rural and underserved areas. Transportation barriers, limited community resources, and insufficient infrastructure continue to hinder participation [15, 42, 43]. These limitations have also been linked to increased social isolation, which is known to elevate mortality risk among older people [7–9]. Gender and socioeconomic status significantly influence SWB outcomes. Women tend to report higher SWB than men, consistent with earlier studies [26, 37]. Conversely, economic constraints restrict older adults' opportunities for participation and may exacerbate frailty, particularly among vulnerable populations [3].

In recent years, digital technologies have reshaped how people interact and engage in meaningful activities. This shift may offer new opportunities for older adults, especially the care-needing groups, to participate in SAP through virtual platforms. Future studies should examine whether digital forms of SAP yield comparable benefits to in-person participation [12, 44, 45].

## Figures and Tables

**Table 1:** Descriptive statistics.

Variable	(i) All		(ii) Healthy		(iii) Care-needing	
	Obs.	Percent (%)	Obs.	Percent (%)	Obs.	Percent (%)
Total	5986	100	5469	100	406	100

*Explained variable (range 1∼3)*						
Subjective well-being (SWB)						
1: Low	428	7.15	367	6.71	50	12.32
2: Medium	2624	43.84	2363	43.21	208	51.23
3: High	2934	49.01	2739	50.08	148	36.45

*Dependent variable*						
Participation frequency (frequency of SAP: frequent participation = 1)	3311	61.57	3100	62.64	176	48.09
The number of activity types (there are more than two for activity types = 1)	941	23.54	907	24.50	32	12.03
Economic status ( good economy = 1)	471	7.66	448	8.00	20	4.63
Single (= 1)	1247	20.60	1077	19.50	135	31.99
Married (= 1)	3834	63.33	3555	64.38	216	51.18
Female (= 1)	3482	56.33	3099	55.11	284	65.59
The super-aged (age 75 and above)	3149	50.95	2698	47.98	352	81.29
Hold housing (with a house = 1)	4785	78.00	4399	78.76	301	70.49
IADL (lowering = 1)	834	13.53	615	10.96	195	45.35
Channels for obtaining information (more than one = 1)	3764	60.90	3525	62.69	193	44.57
“Are you conscious about maintaining and improving your health?” (= 1)	5263	86.73	4824	87.00	344	83.50
Good neighborly relations (has mutual assistance = 1)	3017	50.21	2789	50.07	218	50.82

**Table 2:** Regression results of OLS and ordered probit models.

Variables	Model (1)	Model (2)	Model (3)	Model (4)	Model (5)	Model (6)
	(i) Healthy	(ii) Care-needing	(i) Healthy		(ii) Care-needing	
	OLS (SWB)		Order probit (SWB)			
Participate frequency	0.088^∗∗∗^	0.002	0.202^∗∗∗^	0.168^∗∗∗^	0.101	0.008
The number of activity types	0.074^∗∗∗^	0.232		0.167^∗∗∗^		0.414
Economic status	0.293^∗∗∗^	0.183	0.782^∗∗∗^	0.711^∗∗∗^	0.341	0.353
Single	−0.188^∗∗∗^	0.004	−0.320^∗∗∗^	−0.354^∗∗∗^	0.055	0.011
Married	0.055^∗∗^	0.09	0.087	0.116^∗∗^	0.105	0.166
Female	0.059^∗∗∗^	0.187^∗∗^	0.153^∗∗∗^	0.116^∗∗∗^	0.330^∗∗^	0.337^∗∗^
The super-aged (over age 75)	0.054^∗∗^	0.182	0.075^∗∗^	0.103^∗∗^	0.377^∗∗^	0.328
Hold housing	0.112^∗∗∗^	0.129	0.238^∗∗∗^	0.209^∗∗∗^	0.292	0.236
IADL	−0.119^∗∗∗^	0.099	−0.185^∗∗∗^	−0.227^∗∗∗^	−0.05	0.176
BMI	0.008	0.032	0.015	0.015	0.03	0.051
BMI^2^	−0.000	0	0	0	0	0
Good neighborly relations	0.161^∗∗∗^	0.003	0.362^∗∗∗^	0.327^∗∗∗^	0.087	−0.001
Threshold 1			−0.742^∗∗∗^	−0.769^∗∗∗^	0.223	0.719
Threshold 2			0.882^∗∗∗^	0.845^∗∗∗^	1.839^∗∗^	2.33
Observations	3469	241	4614	3469	325	241
*R*-squared	0.107	0.079				

*Note:* Statistical significance is indicated by ^∗∗∗^*p* < 0.01 and ^∗∗^*p* < 0.05. Threshold 1 and Threshold 2 are the estimated cutoff points separating levels of subjective well-being in the ordered probit model.

**Table 3:** Results of marginal effects.

Subjective well-being (SWB) 1∼3	Marginal effects results			
	(i) Healthy		(ii) Care-needing	
	Model (3)	Model (4)	Model (5)	Model (6)
*a. Participation frequency*				
1. _Predict	−0.024^∗∗∗^	−0.021^∗∗∗^	−0.019	−0.015
2. _Predict	−0.051^∗∗∗^	−0.041^∗∗∗^	−0.017	−0.011
3. _Predict	0.075^∗∗∗^	0.062^∗∗∗^	0.036	0.026

*b. The number of activity types*				
1. _Predict		−0.019^∗∗∗^		−0.071
2. _Predict		−0.042^∗∗∗^		−0.08
3. _Predict		0.062^∗∗∗^		0.151

*c. Economic status*				
1. _Predict	−0.055^∗∗∗^	−0.057^∗∗∗^	−0.054	−0.06
2. _Predict	−0.220^∗∗∗^	−0.200^∗∗∗^	−0.073	−0.069
3. _Predict	0.275^∗∗∗^	0.254^∗∗∗^	0.126	0.13
*N*	4614	3469	325	241
Pseudo *R*^2^	0.063	0.064	0.038	0.042

*Note:* Table 3 reports marginal effects from ordered probit models examining the relationship between SAP and subjective well-being (SWB). Columns (i) and (ii) correspond to healthy and care-needing older adults. Dependent variables include the following: a. participation frequency, b. number of activity types, and c. economic status. Values 1. _predict to 3. _predict indicate predicted probabilities of low, medium, and high SWB. Coefficients indicate the marginal change in the probability of being classified into each SWB category when compared to the reference group (e.g., those with lower levels of participation). Positive values imply higher likelihood of being in that category, and negative values imply lower likelihood. Statistical significance: ^∗∗∗^*p* < 0.01 and ^∗∗^*p* < 0.05. Sample sizes vary due to missing data.

**Table 4:** Marginal effects of activity types on SWB.

Full sample activity type	Marginal effects of activity types on SWB		
	SWB		
	Pr (1 = low)	Pr (2 = medium)	Pr (3 = high)
1. Group of volunteers	−0.006	−0.011	0.018
2. Sports groups or clubs	−0.019^∗∗∗^	−0.034^∗∗∗^	0.053^∗∗∗^
3. Groups with related interests	−0.026^∗∗∗^	−0.047^∗∗∗^	0.073^∗∗∗^
4. Learning/cultural circle	−0.001	−0.001	0.002
5. Long-term disability prevention places	−0.008	−0.015	0.024
6. Senior citizens club	−0.009	0.017	−0.262
7. Town council/autonomous council	−0.020	−0.036	0.056
8. Gainful employment	−0.019^∗∗∗^	−0.035^∗∗∗^	0.055^∗∗∗^

*Note:* Marginal effects are based on ordered probit estimates using the full sample. Values represent the change in the probability of being in each subjective well-being (SWB) category associated with participation in each activity type, holding other variables constant. *N* = 3702; pseudo *R*^2^ = 0.061.

^∗∗^ *p* < 0.05.

^∗∗∗^ *p* < 0.01.

**Table 5:** Regression results of 2SLS.

Variables	Model (7-1)	Model (7-2)	Model (8-1)	Model (8-2)
	(i) Healthy		(ii) Care-needing	
	SAP	SWB	SAP	SWB
Participate frequency		1.617^∗∗∗^		0.911^∗∗^
(a) Number of information sources	0.089^∗∗∗^		0.147^∗∗∗^	
(b) Health consciousness	0.108^∗∗∗^		0.213^∗∗∗^	

*The number of activity types*				
Economic status	0.132^∗∗∗^	0.095	0.113	0.056
Single	0.052^∗∗^	−0.224^∗∗∗^	0.129	−0.076
Married	0.049^∗∗∗^	−0.032	0.092	−0.015
Female	0.004	0.056	−0.045	0.217^∗∗^
The super-aged (over age 75)	−0.072^∗∗∗^	0.140^∗∗∗^	−0.023	0.221^∗∗^
Hold housing	0.043^∗∗^	0.043	0.019	0.135
IADL	−0.134^∗∗∗^	0.151^∗∗^	−0.286^∗∗∗^	0.254
BMI	0.007	−0.004	−0.013	0.028
BMI^2^	−0.000	0	0.000	0
Good neighborly relations	0.126^∗∗∗^	−0.043	0.089	−0.055
Constant	0.271^∗∗∗^	1.391^∗∗∗^	0.449	0.845
First-stage F-statistic	18.7		11.5	
Observations	4595	4595	322	322

*Note:* Column (7-1) corresponds to the healthy older adults (care = 0), and column (8-1) corresponds to the care-needing older adults (care = 1). The dependent variable is the frequency of social and activity participation (SAP). “(a) Number of information sources” and “(b) health consciousness” are the excluded instruments. All standard errors are robust to heteroskedasticity. First-stage F-statistics for both subsamples exceed the conventional threshold of 10. Kleibergen–Paap rk LM tests reject the null hypothesis of underidentification (*p* < 0.001), and Hansen J statistics suggest the overidentifying restrictions are valid (*p* = 0.872 for healthy adults; *p* = 0.586 for care-needing older adults). Statistical significance is indicated by ^∗∗∗^*p* < 0.01 and ^∗∗^*p* < 0.05.

**Table 6:** The frequency of social and activity participation among older adults.

Social and activity participation (SAP)	Low frequency: 0		High frequency: 1			
To participate in frequency	Not to attend	A few times a year	1∼3 times a month	Once a week	Two to three times a week	More than 4 times a week
Group of volunteers	1	2	3	4	5	6
Sports group or club	1	2	3	4	5	6
Groups with related interests	1	2	3	4	5	6
Learning/cultural circle	1	2	3	4	5	6
Long-term disability prevention places (such as busy salons and gymnastics)	1	2	3	4	5	6
Senior citizens club	1	2	3	4	5	6
Town council/autonomous council	1	2	3	4	5	6
Gainful employment	1	2	3	4	5	6

**Table 7:** The type of social and activity participation among older adults.

	0	1	Total	NA (no answer)	0 (%)	1 (%)	NA (%)
Group of volunteers	4009	441	4450	1731	64.86	7.13	28.01
Sports groups or clubs	3391	1244	4635	1546	54.86	20.13	25.01
Groups with related interests	3308	1393	4701	1480	53.52	22.54	23.94
Learning/cultural circle	3910	418	4328	1853	63.26	6.76	29.98
Long-term disability prevention places (such as busy salons and gymnastics)	4009	426	4435	1746	64.86	6.89	28.25
Senior citizens club	4132	256	4388	1793	66.85	4.14	29.01
Town council/autonomous council	4021	415	4436	1745	65.05	6.71	28.23
Gainful employment	3338	1133	4471	1710	54.00	18.33	27.67

## Data Availability

The data used in this study were obtained through a formal application and approval process from the survey data of Sakai City, Osaka, Japan. The sharing of research data is restricted and requires permission from the relevant authorities.

## Nomenclature

SAP: Social and activity participation
SWB: Subjective well-being
LTCI: Long-Term Care Insurance
OECD: Organization for Economic Co-operation and Development
IV: Instrumental variable
OLS: Ordinary least squares
2SLS: Two-stage least squares
IADL: Instrumental activities of daily living
BMI: Body mass index
MHLW: Ministry of Health, Labor and Welfare
PC: Personal computer

## Appendix A. Frequency and Types of SAP Among Older Adults

This appendix provides a detailed description of the types and frequency of SAP used in this study. Eight types of social and activity engagement were assessed: (1) volunteer groups, (2) sports groups or clubs, (3) hobby or interest groups, (4) learning or cultural circles, (5) long-term disability prevention programs (e.g., salon activities or gymnastics), (6) senior citizens' clubs, (7) neighborhood associations or autonomous councils, and (8) gainful employment. Respondents were asked how often they participated in each activity type, with responses categorized into six frequency levels (see Table A1):

1. Not at all
2. A few times a year
3. 1–3 times a month
4. Once a week
5. Two to three times a week
6. More than four times a week

For the purposes of analysis, each activity was recoded into a binary variable: low frequency (0) for those who participated less than once a week (responses 1–3) and high frequency (1) for those who participated once a week or more (responses 4–6). Table A1 presents the original frequency scale, and Table A2 shows the distribution of participation (0 = low frequency; 1 = high frequency) for each activity type, including the number of nonresponses (NA). Prior to constructing the participation frequency variables, we conducted an internal check to explore the possible reasons behind the nonresponse pattern. Specifically, we examined the raw responses to Question 6 (1) of the survey, which asked about engagement in the eight activity types. We found that only 77 respondents out of the full sample of 6181 did not report participation in any activity type, corresponding to a response rate of approximately 98.8%. This suggests that most missing values occurred because respondents were instructed to answer only the items relevant to their actual experiences. As such, the missing responses are unlikely to reflect systematic nonresponse or introduce substantial selection bias into the analysis.
